# Exogenous 2,4-Epibrassinolide Treatment Maintains the Quality of Carambola Fruit Associated With Enhanced Antioxidant Capacity and Alternative Respiratory Metabolism

**DOI:** 10.3389/fpls.2021.678295

**Published:** 2021-06-04

**Authors:** Xiaoyang Zhu, Yuxin Chen, Junyi Li, Xiaochun Ding, Shuangling Xiao, Silin Fan, Zunyang Song, Weixin Chen, Xueping Li

**Affiliations:** ^1^Guangdong Provincial Key Laboratory of Postharvest Science of Fruits and Vegetables/Engineering Research Center for Postharvest Technology of Horticultural Crops in South China, College of Horticulture, South China Agricultural University, Guangzhou, China; ^2^College of Land Resources and Environment, Jiangxi Agricultural University, Nanchang, China

**Keywords:** carambola fruit, brassinolide, fruit quality, respiratory metabolism, antioxidant capacity

## Abstract

Brassinosteroids act by delaying fruit ripening. The effects of different concentrations of 2,4-epibrassinolide (eBL) treatments on carambola fruit ripening were investigated. The results show that treatment of 2.8 mg L^–1^, eBL with 10 min effectively delays ripening and maintains the quality of carambola fruit. This is achieved by retarding color changes and firmness losses while maintaining high level of soluble protein content and vitamin C, and low organic acid content. eBL-delayed senescence may be due to the inhibition of respiration rate and enhanced antioxidant system. It is noteworthy that eBL treatment markedly reduces the content of fructose-6-phosphate (6-P-F) and enhances the activity of cytochrome oxidase (CCO), and the total activity of glucose-6-phosphate dehydrogenase (G-6-PDH) and 6-phosphate gluconate dehydrogenase (6-PGDH). eBL treatment induces the IAA and GA contents but reduces that of ABA. In general, senescence retardation and quality improvement by eBL treatment may be due to the enhanced antioxidant capacity and altered respiratory pathways.

## Introduction

Carambola (*Averrhoa carambola* L.) fruit, commonly known as star fruit, is popular in Southeast Asian countries and China owing to its nutritional and pharmacological value (Gol et al., 2015). It is an economically important fruit in tropical and subtropical areas and has been cultivated for hundreds of years. The unique star shape and attractive appearance, in addition to its special flavor and nutritional and pharmacological value, provide a considerable market potential as a garnish for salads and drinks. Carambola fruit is a typical non-climacteric fruit (Warren and Sargent, 2011). It is a juicy fruit that has a fragile and succulent pulp with attractive flesh and a distinctive flavor. These fruit are highly perishable and have a high moisture content and susceptibility to mechanical damage and dehydration, which cause extensive postharvest losses and limit their marketability. Thus, it is important to explore simple, effective, and inexpensive processes for its preservation. Different techniques have been reported to extend the shelf-life and maintain the quality of harvested carambola fruit, including low-temperature storage (Ali et al., 2004), 1-methylcyclopropene treatment (Warren and Sargent, 2011), edible coatings (Gol et al., 2015), polyamines (PAs) treatment (Ahmad and Ali, 2019) and methyl jasmonate treatment (Mustafa et al., 2016).

Low-temperature storage and modified atmosphere packaging are the main managements used to control carambola fruit ripening. However, low-temperature storage will cause chilling injury and affect the skin coloration and structure of carambola fruit (Ali et al., 2004). The use of a controlled atmosphere could effectively maintain fruit quality and extend the shelf life of fruits and vegetables, but these techniques are capital intensive and expensive and may cause an unpleasant flavor (Kader et al., 1989).

Plant bioregulators are natural and safe phenolic compounds that exhibit a high potential to control postharvest losses of horticultural crops. Among the different bioregulators that have been tested, gibberellic acid, 1-naphthalene acetic acid (NAA), 6-benzylamino purine, and salicylic acid, NAA effectively maintains the quality of carambola fruit, reduces postharvest losses, and prolongs fruit shelf life (Hazarika and Tariang, 2019). Brassinosteroids (BRs) are a class of natural plant hormones that play important roles in regulating plant development, growth, and stress resistance (Bajguz and Hayat, 2009). BRs have been widely used to improve plant stress resistance (Bajguz and Hayat, 2009). As plant growth regulators that have low toxicity and are ecofriendly, BRs have the potential to improve quality of postharvest horticultural products. Postharvest BR treatment effectively delayed jujube fruit senescence by reducing ethylene production, effectively inhibiting the development of blue mold rot by enhancing activities of defense-related enzymes and maintaining fruit quality (Zhu et al., 2010). Other studies showed that BRs induced fruit ripening via increasing ethylene production (Zhu T. et al., 2015). For example, treatment with BRs promoted mango fruit ripening *via* an acceleration of the ethylene production and respiration rate (Zaharah et al., 2012). BRs promoted persimmon fruit ripening by promoting ethylene production, increasing the respiration rate, and influencing cell wall-degrading enzymes and ethylene biosynthesis (He et al., 2018). Brassinolide application effectively induced tomato fruit ripening by increasing soluble sugars, ascorbic acid, lycopene contents, respiration rate, and ethylene production, as well as the expression of genes related to ethylene synthesis (Zhu T. et al., 2015).

To date, the effects of BRs on carambola fruit are not known. The aim of this study was to investigate the effects of BRs on postharvest fruit ripening and quality attributes of carambola fruit and the possible mechanisms for these effects.

## Materials and Methods

### Plant Material and Treatments

Carambola fruit were collected from an orchard in Zengcheng (Guangzhou, China). The variety named “*Averrhoa carambola* Linn. cv. Xiangmi” was introduced from Singapore. Fruit at approximately 70% maturity were harvested, rapidly transported to the laboratory and precooled at 20°C. The fruit that were cleaned with water and air-dried at room temperature that lacked diseases and blemishes and were of uniform shape, weight, and maturity were selected for further treatment.

For the preliminary experiments, the selected carambola fruit were randomly divided into four groups, and each group contained 80 fruit. The 2,4-epibrassinolide (eBL) (EKEAR Biotechnology Co., Ltd., Shanghai, China) were dissolved in ethanol and then diluted in distilled water (treatment solution) as the desired concentrations. Four concentration of eBL treatment solution, including 0, 1.4, 2.8, and 9.4 mg L^–1^, were prepared, and fruit were immersed in different eBL solutions for 10 min, and then air-dried for 2 h. The carambola fruit were placed in unsealed plastic bags (0.02 mm thick) and stored at room temperature (25 ± 1°C). Fruit ripening process were then monitored by the periodic measurement of respiration, firmness, and peel color.

**FIGURE 1:**
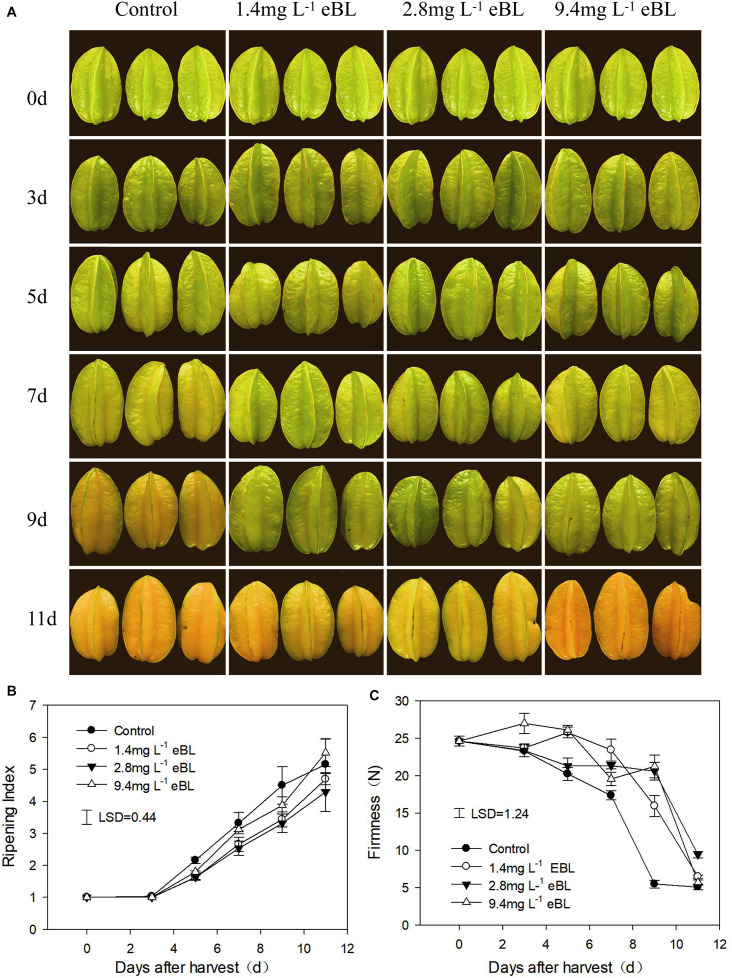
The effects of different 2,4-epibrassinolide (eBL) concentrations on carambola fruit ripening. **(A)** Photos of carambola fruit treated with different concentration of eBL; **(B,C)** fruit ripening index **(B)** and firmness **(C)** under different treatments. Least significant differences (LSDs) were calculated to compare significant effects at the 5% level. Each data point represents the mean ± S.E. (*n* = 3).

Several preliminary experiment results showed that 2.8 mg L^–1^ eBL treatment exhibited the desired effects among the four concentrations tested (Figure 1). Then 2.8 mg L^–1^ eBL treatment was selected to further study the effects of eBL on carambola fruit ripening and quality. Fruit after pretreatment were then selected randomly and divided into two groups, and each group contained 120 fruit. Fruit in each group were soaked in a water solution with 0 and 2.8 mg L^–1^ eBL for 10 min, respectively, and then air-dried for 30 min and stored at 25 ± 1°C. The carambola fruit were placed in unsealed plastic bags (0.02 mm thick). At least 15 fruit were monitored by the periodic measurement of respiration, firmness, and peel color, respectively. Samples were collected at 0, 3, 5, 7, 9, and 11 days. The middle part of fruit without seeds was collected and diced, frozen in liquid nitrogen, and stored at −80°C for further use. All the treatments were conducted in three biological replicates.

### Evaluation of Fruit Ripening

The fruit ripening was evaluated and monitored by periodic measurements of fruit respiration, firmness, and peel color. Fruit color was evaluated by manual observation and measurement with a colorimeter. Manual observations were evaluated by the fruit ripening index, which is a scale from 1 to 6 as described by Omar et al. (2012) with minor modifications: (1) Bright green in color, green-mature; (2) green with a small amount of yellowish color; (3) more green than yellow; (4) more yellow than green; (5) Yellow with a small amount of green; and (6) Completely yellow. The fruit color was also determined using a chromameter-2 reflectance colorimeter (Minolta, Osaka, Japan) equipped with a CR-300 measuring head and recorded as lightness (*L*), hue angle (*h*), or chroma (*c*). Five points around the equatorial region on each fruit were selected for the measurement. The fruit firmness was determined using an Instron 5,542 penetrometer (Instron, Norwood, MA, United States) equipped with a cylindrical flat-surfaced plunger (5 mm diameter). A 2-mm slice of fruit skin was removed, and the firmness of fruit was measured at a penetration depth of 2 cm on three different fruit at five different points per fruit. Fruit firmness (N) was expressed as the mean of these 50 measurements. Fruit respiration was determined using gas chromatograph as described by Zhu X. et al. (2015).

### Determination of Soluble Protein and Vitamin C and Soluble Protein Contents

Fruit flesh (100 g) was cut into small pieces, homogenized, and filtered to measure the ascorbic acid (AsA) contents as described by Lu et al. (2010). The soluble protein was determined as described by Deng et al. (2012). One gram of pulp was ground with 4 ml extraction buffer [0.05 mol L^–1^ phosphate buffer, pH 7.8, containing 0.2 mmol L^–1^ EDTA and 1% polyvinylpyrrolidone (PVP)]. The mixture was centrifuged at 13,000 r min^–1^ for 20 min at 4°C. The supernatant was collected as the protein extraction. Then 1 ml of the supernatant was taken and 5 ml of Coomassie Brilliant Blue G-250 solution was added and mixed. The determination was conducted by measuring the absorbance at 595 nm.

### Determination of Sugar and Organic Acid Contents

Glucose, fructose, and sucrose are the main sugar components in fruit, which were measured as described by Wang et al. (2010), with minor modifications. Briefly, 5 g of carambola pulp was ground in liquid nitrogen and then 1 g powder and 50 ml of distilled water were mixed crushed by a cell grinder (BILON96-II, Shanghai, China). The sugar compounds were extracted 60 times by ultrasonic oscillation (ultrasonic time of 3 s, gap time of 5 s, and ultrasonic power of 150 W). Then the sample mixture was filtered with 0.22 μm filter, and the filtrate was used for the determination of soluble sugars. The separations were performed on a Dionex ICS3000 Multifunctional Ion Chromatograph (Dionex, Sunnyvale, CA, United States), using a Dionex CarboPac PA1 (2 × 250 mm) column at 30°C with 40 mmol L^–1^ NaOH as the eluent at a flow rate of 0.25 ml min^–1^.

A standard curve was drawn using the standard solution. The standard solution was prepared as follows: accurately weigh 0.5 g of fructose, glucose, and sucrose and diluted to a 100-ml volumetric flask with ultrapure water to obtain a single standard solution with each concentration of 5.0 mg ml^–1^. At the same time, a mixed standard stock solution of fructose, glucose, and sucrose was prepared, with the concentration of fructose, glucose, and sucrose being all 5.0 mg ml^–1^. The standard solution was gradually diluted for sampling to draw the standard curve.

The organic acid was determined using an Agilent 1200 series rapid-resolution LC system (Agilent Technologies, CA, United States). Briefly, 10 g of pulp sample was ground with a freezer grinder, then 1 g of powder was mixed with the extraction solution (8 ml of 0.015 mol L^–1^ potassium dihydrogen phosphate (pH = 3) with 1.0% HPLC purity of methanol) in a 10-ml tube. The mixture was vortex mix for 30 s and shocked at 40°C for 2 h, and then centrifuged at 11,000 × *g* for 15 min. The supernatant solution was collected and added to 10 ml volume with the extract solution. The final extraction solution was filtered with 0.45 filter, and the filtrate was used for the determination of organic acid.

The determination was conducted using an Agilent 1200 series rapid-resolution LC system plus with an Agilent Eclipse Plus C18 column. The mobile phase is the 0.015-mol L^–1^ potassium dihydrogen phosphate (pH = 3) and 1% HPLC-grade methanol mixture. The column temperature was maintained at 25°C, and the injection volume was 15 μl, with a flow rate at 0.8 ml min^–1^.

### Analysis of Malondialdehyde and H_2_O_2_ Contents and Antioxidant-Related Enzymatic Activities

The malondialdehyde (MDA) content was measured as described by Guo et al. (2013). MDA content was determined by adding 0.6% thiobarbituric acid (TBA) and 2 ml crude extract. The mixture was boiled in a water bath for 15 min and then quickly cooled in an ice bath. The absorbance was measured at 532 nm with UV-2450 spectrophotometer (Shimadzu Corporation, Kyoto, Japan) and corrected for non-specific absorption at 600 nm. The MDA concentration was calculated using the extinction coefficient of 155 mM cm^–1^. H_2_O_2_ was determined using a Hydrogen Peroxide Assay Kit (A064, Nanjing Jiancheng Bioengineering Institute, Nanjing, China) according to the manufacturer’s instructions.

For the enzyme extraction, all procedures were carried out at 4°C. The activities of polyphenol oxidase (PPO) and peroxidase (POD) were measured as described by Shi et al. (2013). Briefly, 1 g of the pulp was ground and homogenized with 4 ml of extraction buffer (0.2 mol L^–1^ sodium phosphate buffer (pH 6.5) containing 0.01 g ml^–1^ PVP for PPO, and 0.2 mol L^–1^ sodium phosphate buffer (pH 6.4) with 0.02 g ml^–1^ PVP for POD). Then the extracts were centrifuged at 12,000 × *g* for 20 min at 4°C, and the supernatants were used as crude enzyme. PPO activity was determined by measuring absorbance at 410 nm. For POD, the reaction mixtures containing 0.5 ml crude extract and 2 ml guaiacol substrate (100 mmol L^–1^ sodium phosphate (pH 6.4) and 8 mmol L^–1^ guaiacol) were incubated for 5 min at 30°C. The increase in absorbance at 460 nm was determined after 1 ml H_2_O_2_ (24 mmol L^–1^) was added.

The catalase (CAT) and superoxide dismutase (SOD) activity were measured as described by You et al. (2004) with a slight modification. Crude enzyme was extracted using 1 g of pulp and 4 ml of extraction buffer containing 0.05 g of polyvinylpolypyrrolidone (PVPP) (50 mmol L^–1^ sodium borate buffer (pH 7.0, containing 5 mmol L^–1^ mercaptoethanol) for CAT, sodium phosphate buffer (100 mM, pH 6.4) was used for SOD. The reaction mixture contained 0.5 ml enzyme, 2 ml sodium phosphate buffer (50 mmol L^–1^, pH 7.0), and 0.5 ml H_2_O_2_ (40 mmol L^–1^) in a total volume of 3.0 ml. The decomposition of H_2_O_2_ was measured by the decline in absorbance at 240 nm.

The activity of ascorbic acid oxidase (AAO) was determined using an AAO Activity Assay Kit (Nanjing Jiancheng Bioengineering Institute) according to the manufacturer’s instructions.

### Determination of the Activities of Respiratory Metabolic Enzymes

Four key enzymes involved in different respiratory metabolism pathways were tested, including phosphate hexose isomerase (PGI), succinate dehydrogenase (SDH), cytochrome oxidase (CCO), and the total activity of glucose-6-phosphate dehydrogenase (G-6-PDH) and 6-phosphate gluconate dehydrogenase (6-PGDH). The PGI activity is directly reflected by the content of substrate fructose 6-phosphate (6-P-F). The PGI activity was measured as described by Li et al. (2016). Briefly, 0.5 g of frozen samples was ground in 2.5 ml Tris-HCl buffer (0.05 mol L^–1^, pH 7.4) on ice and then centrifuged at 5,000 × *g* at 4°C for 30 min. The supernatant was collected and stored at 4°C until use. A volume of 0.5 ml of the supernatant and 1 ml of glucose 6-phosphate (6-P-G) was mixed and incubated in a water bath for 5 min at 30°C, and then 2 ml of trichloroacetic acid was added and centrifuged at 5,000 × *g* at 4°C for 15 min. A volume of 1 ml of supernatant, 6 ml of HCl, and 2 ml of resorcinol were mixed, and the content of 6-P-F content was determined at 520 nm using 6-P-F as a standard. The content of 6-P-F was expressed as grams per kilogram.

The activity of SDH was measured as described by Li et al. (2016) with minor modifications. Briefly, 0.5 g of sample was ground with 2 ml of phosphate buffer (pH 7.2) and centrifuged at 10,000 × *g* at 4°C for 15 min. The precipitate was suspended in 5 ml of Tris-HCl buffer (0.06 mol L^–1^) and used as the enzyme solution. The reaction solution was composed of 0.1 mL of 1.5 mol L^–1^ potassium phosphate buffer (pH 7.4), 0.1 ml of 1.2 mol L^–1^ succinic acid (pH 7.4), 0.1 ml of 0.9 mmol L^–1^ 2,6-sodium dichloroindophenol and 2.5 ml of distilled water. The reaction solution was incubated at 30°C for 10 min, and 0.1 ml of enzyme was added and gently mixed. A volume of 0.1 ml of phenazine methyl sulfate (PMS) was added to the start of the reaction in a cuvette, and the absorbance at 600 nm was recorded using a Powerwave 340 plate reader (Biotek Instruments, Winooski, VT, United States) after 15–60 s of reaction. A change in the OD_600_ per minute of 0.01 was defined as a unit of enzyme activity (U), and the enzyme activity was expressed as in Units per kilogram (FW).

The activity of CCO was determined as described by Li et al. (2016). Briefly, 0.5 g of a frozen sample was ground in 1.25 ml phosphate buffer (0.04 mol L^–1^, pH = 2), shaken for 30 s and then centrifuged at 3,000 × *g* at 4°C for 10 min. The supernatant was collected for enzyme assay. A volume of 0.1 ml of supernatant, 0.2 ml cytochrome *c* (0.04%), and 3 ml of distilled water were preincubated at 37°C for 2 min, and then 0.1 ml of dimethyl-*p*-phenylenediamine (0.4%) was added and incubated at 37°C for 1–3 min. The pH was adjusted to 5.6∼6 using 0.1 mol L^–1^ HCl. A volume of 10 ml of the reaction solution was centrifuged at 5,000 × *g* at 4°C for 5 min, and the absorbance was recorded at 510 nm for 10 min. The CCO activity was calculated as follows: OD × dilution factor/fresh weight and expressed as OD per kilogram FW.

The total activities of G-6-PDH + 6-PGDH were assayed as described by Li et al. (2016). A total of 0.5 g of frozen samples was ground in 2.5 ml of prechilled extraction buffer (0.05 mol L^–1^ phosphate buffer, pH 6.8, 0.25 mol L^–1^ sucrose, 0.005 mol L^–1^ EDTA, and 1 g L^–1^ bovine serum albumin) on ice, centrifuged at 10,000 × *g* at 4°C for 15 min, and the supernatant was discarded. The precipitate was suspended in 2.5 ml of 0.05 mol L^–1^ Tris-HCl buffer (pH 7.4) as the enzyme solution. A volume of 0.05 ml of enzyme solution was added in 0.45 ml reaction solution [5 mmol L^–1^ 6-P-G, 5 mmol L^–1^ MgCl_2_, 5 mmol L^–1^ Tris-HCl (pH 7.4)], and the absorbance was immediately measured at 340 nm. A volume of 0.05 ml 0.1 mol L^–1^ Tris-HCl buffer (pH = 7.4) was used as the control. The total activities of G-6-PDH + 6-PGDH were expressed as millimoles NADP per kilogram per minute.

### Determination of the Content of Endogenous Hormones

Three types of hormones (indole-3-acetic acid (IAA), gibberellin acid (GA_3_), and abscisic acid (ABA)) were analyzed as described by Zhang et al. (2005) with minor modifications. Briefly, 1.0 g of frozen sample was ground in liquid nitrogen, and 8-ml precooled 80% methanol (*v*/*v*) was added, mixed, and extracted at 4°C overnight. After centrifugation (10,000 × *g* at 4°C for 15 min), the supernatant was collected. The pellet was reextracted with 6 ml of prechilled 80% methanol. A total of 0.3 g PVPP was added to the combined supernatants, shaken at 100 g at 4°C for 1 h and centrifuged at 10,000 × *g* at 4°C for 15 min. The supernatants were evaporated to dryness under nitrogen gas, reconstituted in 80% methanol, and filtered (PTFE, 0.22 μm; ANPEL Laboratory Technologies, Shanghai, China) before HPLC.

The extracts were analyzed using an Agilent 1200 Series liquid chromatography system (Agilent Technologies, Santa Clara, CA, United States) equipped with an Agilent Eclipse Plus C18 column. A volume of 10 μl of sample was injected with the mobile phase (100% HPLC-grade methanol, acetonitrile, phosphate acid buffer solution (pH 6) = 20:15:65) at a flow rate of 0.8 ml min^–1^. The column temperature was maintained at 35°C. A diode-array detector (DAD) was used for detection at 210 nm for IAA and GA_3_ and 254 nm for ABA. IAA, GA3, and ABA were identified by comparing their retention volumes with those of reference standards (Sigma-Aldrich, United States).

### Statistical Analysis

The experiment was arranged in a completely randomized design. Each treatment comprised three replicates. The data were analyzed using SPSS 17.0 (SPSS, Chicago, IL, United States). SigmaPlot 10.0 (Systat Software, San Jose, CA, United States) was used to create most of the figures. All the results were expressed as the mean ± S.E.

## Results

### The Effects of eBL on Fruit Ripening

First, we screened a suitable concentration of eBL to use to delay fruit ripening. As shown in Figure 1, three different concentrations were tested, and we observed that low concentrations of eBL (1.4 and 2.8 mg L^–1^) could effectively delay fruit coloring process as compared with the control, particularly for the 2.8-mg L^–1^ treatment, but there was no significant difference between a high concentration (9.4 mg L^–1^) of eBL and the control (Figure 1A). The fruit firmness gradually decreased during storage and dropped rapidly from days 7 to 9. All the eBL treatments delayed the decrease in firmness, and fruit treated with 2.8 mg L^–1^ eBL were more firm compared with the others at end of storage (Figure 1B). These results indicated that treatment with 2.8 mg L^–1^ eBL could effectively delay fruit ripening. Thus, 2.8 mg L^–1^ eBL was selected for further study.

**FIGURE 2:**
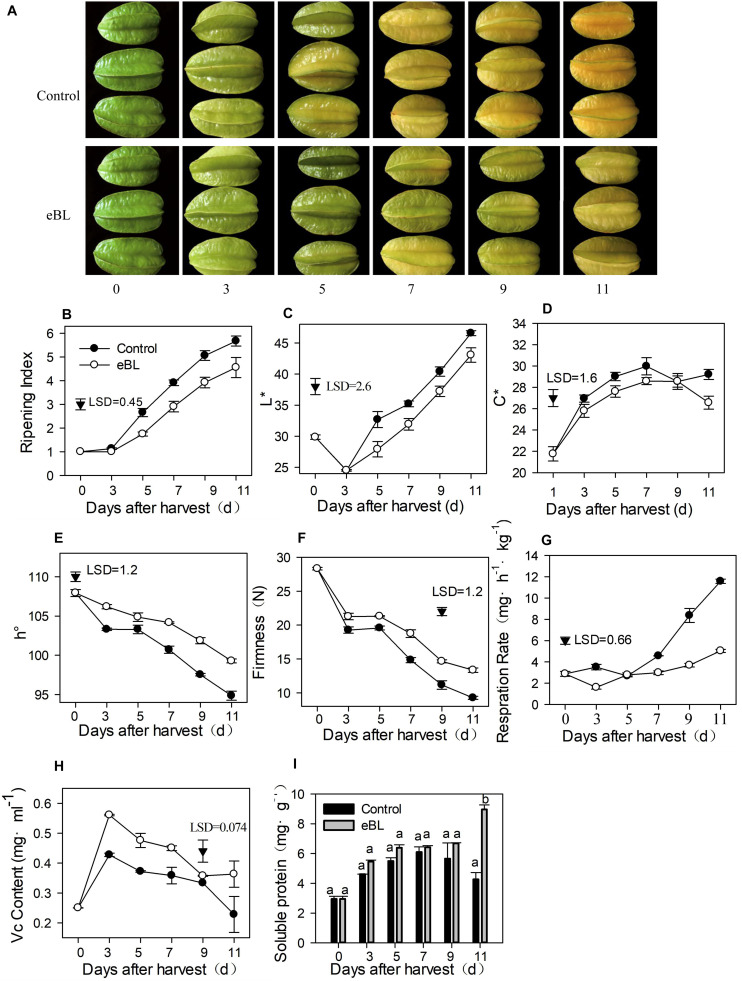
The effects of 2,4-epibrassinolide (eBL) on carambola fruit ripening. **(A)** Photos of carambola fruit treated with or without eBL; **(B)** fruit ripening index assessment; **(C–E)** fruit color changes in L* value **(C)**, C* value **(D)**, and *h*° value **(E)**; **(F)** fruit firmness changes; **(G)** fruit respiration rate; **(H,I)** the content of Vc **(H)** and soluble protein **(I)**. Carambola fruit were stored at room temperature (25 ± 1°C) after treatment. Least significant differences (LSDs) were calculated to compare significant effects at the 5% level. Each data point represents the mean ± S.E. (*n* = 3). Means within a column among different groups followed by the same letter are not significantly different at the 5% level.

As shown Figure 2, eBL treatment (2.8 mg L^–1^) effectively delayed the coloring process of carambola fruit by approximately 2 days (Figures 2A,B). Color is one of the most important indicators of fruit ripening and quality. The color was normally measured using the values of *L*^∗^, *C*^∗^ and *h*°, which indicated the brightness, color saturation *C*^∗^, and chromaticity angle, respectively. *L*^∗^ indicates brightness, and *C*^∗^ indicates the brightly colored fruit. The larger the value, the brighter the fruit color, while *h*° is the characteristic value of color, indicating the hue of different colors. The *h*° of carambola from preharvest to full ripening of fruit ranged from 90 to 110°, and the value was closer to 90, indicating that the carambola was yellow. The *L*^∗^ decreased during the first 2 days and then increased gradually from day 3 in control fruit. The *C*^∗^ value gradually increased as the fruit ripened. Treatment with eBL delayed the increase in *L*^∗^ and *C*^∗^ values compared with those of the control (Figures 2C,D). The *h*° decreased with fruit ripening, and the eBL treatment delayed the decrease (Figure 2E). All these data indicated that the eBL treatment effectively delayed changes in fruit color. The eBL treatment also maintained fruit firmness and reduced its respiration rate compared with the control (Figures 2F,G).

The Vc content changed in a similar manner in eBL-treated and control fruit with an increase during the first 3 days but a gradual decrease during the latter storage; however, the Vc content was much higher in eBL-treated fruit than in the control fruit (Figure 2H). The content of soluble protein increased during storage before slightly decreasing at the latter storage in the control group. The eBL treatment induced production of soluble protein, which increased with storage and was much higher than that in the control fruit (Figure 2I).

### The Effect of eBL Treatment on Content of Sugars and Organic Acids

**FIGURE 3:**
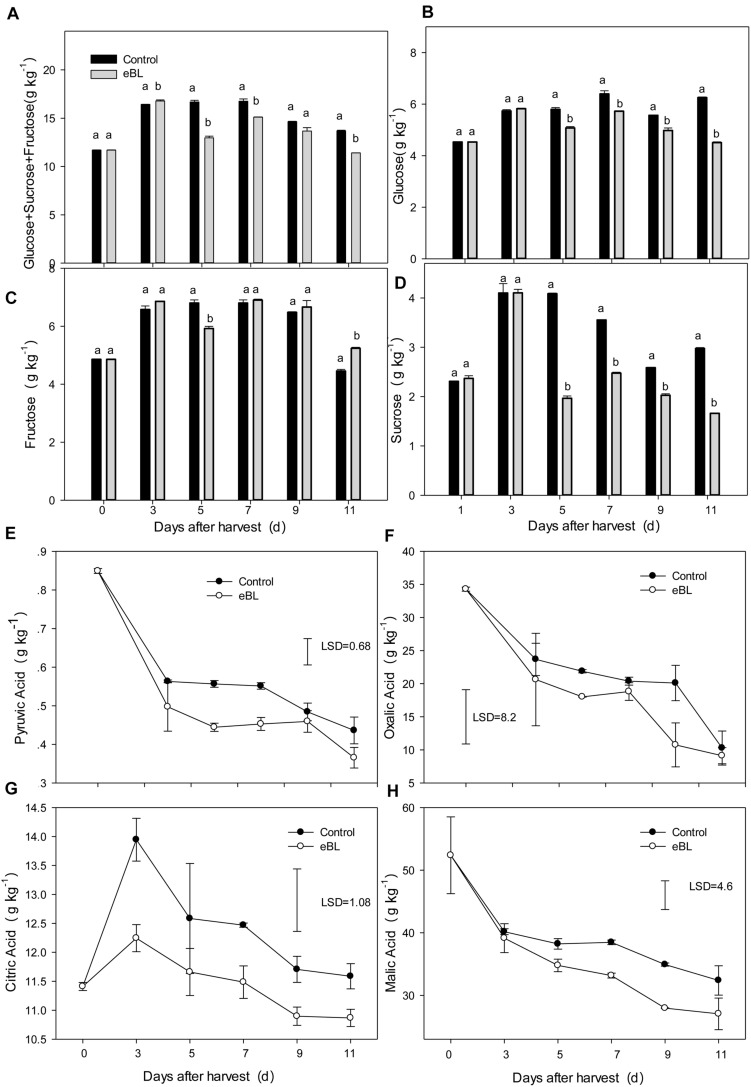
Effects of eBL treatment on the content of sugars and organic acids. **(A)** The total content of glucose, fructose, and sucrose; **(B–D)** the total content of glucose **(B)**, fructose **(C)**, and sucrose **(D)**; **(E–H)** the total content of pyruvic acid **(E)**, oxalic acid **(F)**, citric acid **(G)**, and malic acid **(H)**. Least significant differences (LSDs) were calculated to compare significant effects at the 5% level. Each data point represents the mean ± S.E. (*n* = 3).

Sugar and organic acid contents are the most important indicators of fruit quality. As shown in Figure 3, treatment with eBL significantly altered the accumulation of sugars and organic acids, particularly for the organic acids (Figure 3). The three main sugars in carambola increased with fruit storage and then decreased from day 7. The eBL treatment slightly reduced the total sugar content at days 5 and 11, particularly for the content of sucrose (Figures 3A–D). Four main types of organic acids, including pyruvic acid, oxalic acid, citric acid, and malic acid were tested. The contents of pyruvic acid, oxalic acid, and malic acid decreased with storage, and treatment with eBL enhanced the decrease, which was much lower in the eBL-treated fruit than that of the control fruit (Figures 3E,F,H). Only the content of citric acid showed a sharp increase during the first 3 days before gradually decreasing during the latter storage. Treatment with eBL reduced the content of citric acid compared with that of control (Figure 3G).

### The Effect of eBL Treatment on the Antioxidant Capacity of Fruit

**FIGURE 4:**
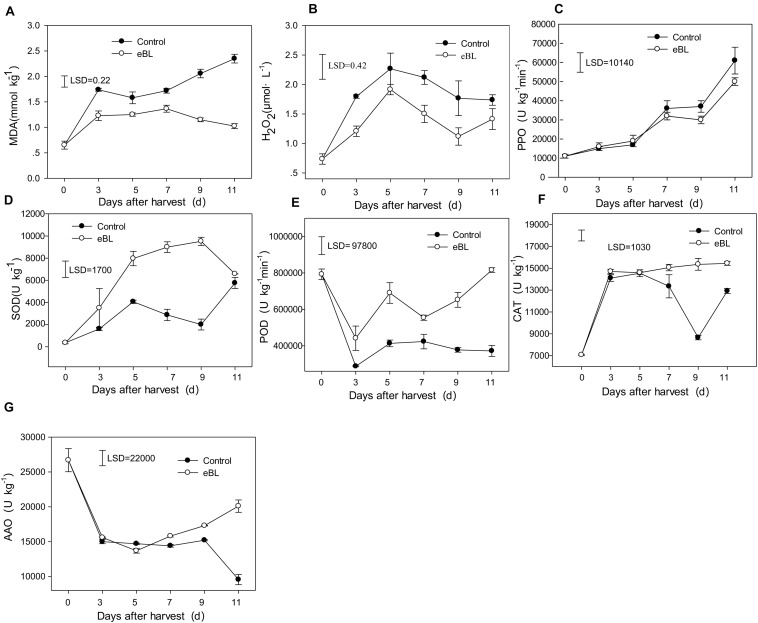
Effects of eBL treatment on the content of reactive oxygen species and antioxidant-related enzyme activities. **(A)** The content of MDA; **(B)** the content of H_2_O_2_; **(C)** PPO activity; **(D)** SOD activity; **(E)** POD activity; **(F)** CAT activity; **(G)** AAO activity. Least significant differences (LSDs) were calculated to compare significant effects at the 5% level. Each data point represents the mean ± S.E. (*n* = 3).

MDA is the end product of cellular lipid peroxidation, and its content can indicate the extent of lipid peroxidation. As shown in Figure 4A, the content of MDA increased with fruit storage in the control fruit. Treatment with eBL dramatically reduced the content of MDA in carambola fruit, which increased slightly and remained stable during the latter storage period (Figure 4A), indicating that eBL alleviated the degree of cell membrane lipid peroxidation and reduced cell membrane damage. The content of H_2_O_2_ in the control fruit increased rapidly during the first 5 days and then decreased slightly during the latter storage period (Figure 4B). Similar changes were observed in the eBL-treated fruit, but the content was much lower in the eBL-treated fruit.

The activities of four antioxidant enzymes were tested to determine the antioxidant system. As shown in Figure 4C, the activity of PPO gradually increased with storage; treatment with eBL slightly reduced the activity of PPO compared with that of the control, but no significant difference was observed (Figure 4C). The activity of SOD increased during the first 3 days and then decreased slightly, but it increased again from day 9. Treatment with eBL dramatically induced the activity of PPO, which increased rapidly during the first 9 days and then decreased slightly. The PPO activity was significantly higher in eBL-treated fruit than that of control (Figure 4D). The activity of POD dropped sharply during the first 3 days and then increased slightly and remained stable during later storage. Similar changes were observed for eBL-treated fruit, but eBL significantly induced the activity of POD (Figure 4E). The activity of CAT in the control fruit increased sharply during the first 3 days and the decreased with storage, but it increased again at the end of storage. Following the treatment with eBL, the activity of CAT dramatically increased during the first 3 days compared with control and then remained stable during the latter storage period, resulting in much higher activity than that in the control group (Figure 4F). The activity of AAO decreased sharply during the first 3 days, then remained at a stable level and decreased from the days 9 to 11. There was no significant difference between the control and eBL treatment from 0 to 9 days, but the AAO activity was significantly higher during latter storage period in eBL-treated fruit compared with that in the control fruit (Figure 4G).

### The Effect of eBL Treatment on the Activities of Respiratory Metabolic Enzymes

**FIGURE 5:**
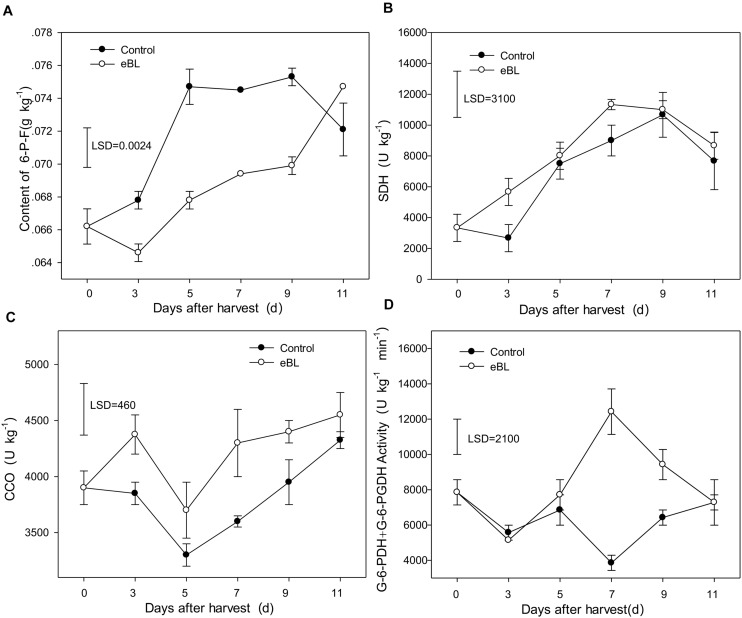
Effects of eBL treatment on the activities of respiration-related enzymes. **(A)** The content of 6-P-F; **(B)** SDH activity; **(C)** CCO activity; **(D)** the total activities of G-6-PDH + 6-PGDH. Least significant differences (LSDs) were calculated to compare significant effects at the 5% level. Each data point represents the mean ± S.E. (*n* = 3).

PGI is a key enzyme in the glycolytic [Embden-Meyerhof-Parnas (EMP)] pathway. Fructose-6-phosphate (F-6-P) is the product catalyzed by PGI in EMP respiratory pathway, which demonstrates the activities of PGI. As shown in Figure 5A, the content of F-6-P increased gradually as fruit ripened. Compared with the control group, treatment with eBL significantly reduced the content of F-6-P, indicating that the activity of PGI was inhibited. SDH is a key enzyme in the tricarboxylic acid (TCA) cycle. As shown in Figure 5B, the activity of SDH increased gradually as fruit ripened and reached its peak on day 9 before decreasing. Compared with the control, treatment with eBL slightly induced the activity of SDH although the increase was not statistically significant (Figure 5B). CCO is a cytochrome oxidase and key enzyme in the cytochrome pathway (CCP). CCO activity decreased during the first 5 days and then increased from days 5 to 11 (Figure 5C). Treatment with eBL induced the activity of CCO during storage.

G-6-PDH and 6-PGDH are the key enzymes of the pentose phosphate pathway (PPP). Their activities are directly related to the PPP pathway. Compared with the control, treatment with eBL induced the activities of G-6-PDH and 6-PGDH. The activities of G-6-PDH and 6-PGDH remained relatively stable during storage, and no significant difference was identified during the first 5 days between the control and samples treated with eBL. The enzymes in fruit treated with eBL rapidly increased from day 5 and reached their maximal levels of activity on day 7, which was significantly higher than those of the control, and then decreased (Figure 5D). These results showed that treatment with eBL reduced the EMP pathways and strengthened the respiratory metabolic pathway of CCO and PPP during storage of carambola fruit.

### The Effect of Treatment With eBL on Contents of IAA, GA3, and ABA

**FIGURE 6:**
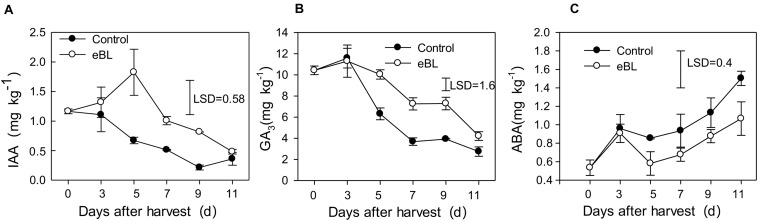
Effects of eBL treatment on the content of endogenous hormones. **(A)** IAA; **(B)** GA3; **(C)** ABA. Least significant differences (LSDs) were calculated to compare significant effects at the 5% level. Each data point represents the mean ± S.E. (*n* = 3).

As shown in Figure 6, the IAA content decreased during fruit storage, but treatment with eBL induced its accumulation, which increased during the first 5 days and then gradually decreased. The content of IAA in fruit treated with eBL was significantly higher than that of the control fruit (Figure 6A). The content of GA_3_ remained at a relatively stable level for the first 3 days and then decreased gradually with fruit ripening. Similar changes in the content of GA_3_ were observed for fruit treated with eBL, but the content of GA_3_ in fruit treated with eBL was much higher than that of control fruit (Figure 6B). ABA increased with fruit ripening, and fruit treated with eBL exhibited a lower-level ABA compared with that of the control fruit (Figure 6C).

## Discussion

Extensive studies over the past three decades have revealed that BRs are important regulators for plant development, growth, and adaption to stress (Bajguz and Hayat, 2009). The potential role of BRs in regulating fruit ripening has also been investigated in recent decades. Most of these studies focus on their roles of maintaining the quality of fruit sensitive to chilling injury. For example, treatment with BRs has been proven to enhance the chilling tolerance in harvested tomato (Aghdam et al., 2012), bamboo shoots (Liu et al., 2016), and mango (Li et al., 2012), which primarily act by enhancing the activities of enzymes involved in antioxidant pathways and those related to proline and energy metabolism and membrane integrity. In this study, we showed that treatment with eBL delayed the ripening of carambola by inhibiting color changes, decreasing firmness, and reducing respiration of fruit. Treatment with eBL also improved the flavor of fruit by maintaining content of vitamin C and reducing content of organic acids. Similar results have also been reported in jujube fruit, in which treatment with BR effectively delayed the development of senescence by reducing production of ethylene (Zhu et al., 2010). BR effectively reduced the respiration of fruit and enhanced activities of antioxidant enzymes in carambola and jujube fruit. In peach fruit, BR treatment reduced fruit decay caused by *Penicillium expansum* associated with the induction of antioxidant activity. BR also delayed fruit senescence *via* reducing the decrease in flesh hardness, content of soluble solids (SSC), and titratable acid (TA) in peach fruit (Ge et al., 2016). Recent work showed that eBR treatment delay apple and pear fruit ripening by suppressing ethylene biosynthesis. BR activated the expression of PuBZR1, a key transcription factor in BR signaling pathway, which interact with PuACO1 and suppresses expression of *ACO1* and *ACS1a* and ACO1 activity, thereby reducing ethylene production during pear and apple fruit ripening (Ji et al., 2020). In strawberry, preharvest eBL treatment significantly enhanced strawberry fruit nutritional and overall quality *via* enhancing fruit total antioxidant activity, total phenolics, and total anthocyanins contents and reducing the decay extension and microbial count during storage. The effects of eBL also showed dose dependence which low concentration showed better effect than high concentration (Sun et al., 2019; Zahedipour-Sheshglani and Asghari, 2020). These results are also in line with our results that suitable low concentration of eBL showed better effect on maintaining carambola than high concentration (Figure 1).

However, a substantial amount of research showed that BRs play dual effects on fruit ripening, which also accelerated fruit ripening in a number of types of fruit, including tomato (Zhu T. et al., 2015), mango (Zaharah and Singh, 2012), and persimmon (He et al., 2018). Treatment with BRs promoted ripening in mango fruit *via* accelerating the production of ethylene and increasing rate of respiration (Zaharah and Singh, 2012). Treatment with BRs promoted ripening in persimmon fruit by promoting the production of ethylene, increasing respiration rate, and influencing cell wall-degrading enzymes and ethylene biosynthesis (He et al., 2018). The application of brassinolide effectively induced ripening in tomato fruit by increasing soluble sugars, ascorbic acid, contents of lycopene, respiration rate, and ethylene production, as well as the expression of genes related to ethylene synthesis (Zhu T. et al., 2015). These results indicated that BRs may play a different role in various fruit species, depending on their ripening process. Other treatments also effectively function to preserve carambola fruit. For example, treatment with polyamines (PAs) effectively maintains the quality of postharvest carambola fruit by enhancing the capacity of antioxidants and reducing the activity of cell wall-degrading enzymes, thus prolonging the shelf life of fruit (Ahmad and Ali, 2019). Treatment with methyl jasmonate improved the quality of carambola fruit and alleviated chilling injury during cold storage and transportation (Mustafa et al., 2016). Treatment with edible coatings significantly delayed the senescence of carambola fruit and maintained fruit quality (Gol et al., 2015). However, to our knowledge, this is the first report that a simple eBL treatment could effectively delay carambola fruit senescence and maintain fruit quality. BRs could be a potential commercial application in the carambola industry owing to their safe nature and simple operation.

The process of fruit ripening has been considered an oxidative phenomenon that requires a turnover of active oxygen species (AOS), including H_2_O_2_ and the superoxide anion (Kaushal, 2016). It has been reported that the formation of O_2_^–^ and accumulation of H_2_O_2_ increased significantly during fruit ripening, as indicated by an increase in protein oxidation and lipid peroxidation products (Jimenez et al., 2002). Therefore, the antioxidant systems play an important role in both fruit ripening and senescence processes (Jimenez et al., 2002). Changes in the antioxidant systems during fruit ripening have previously been described. For example, Jiménez et al. reported that the levels of H_2_O_2_ and lipid peroxidation increased with fruit ripening, as well as the antioxidant components: glutathione (GSH) and AsA content and antioxidant enzyme activities of CAT, SOD, and ascorbate peroxidase (APX) in tomato (Jimenez et al., 2002). However, in blackberry fruit, it was shown that GSH, AsA, and related enzymes declined with fruit ripening (Wang and Jiao, 2001). In jujube fruit, the total antioxidant components increased with fruit ripening (Zhu et al., 2010). In this study, the H_2_O_2_ content increased with fruit ripening, accompanied with the lipid peroxidation level that was indicated by an increase in the content of MAD (Figure 4). The antioxidant component Vc increased at first and then slightly decreased with fruit ripening. The enzymatic activities of PPO, SOD, and CAT increased with fruit ripening, but those of POD and AAO declined with fruit ripening (Figure 4).

In this study, eBL treatment significantly reduced the contents of H_2_O_2_ and MDA by enhancing the activities of PPO, SOD, POD, CAT, and AAO, resulting in a lower degree of oxidative damage. Treatment with eBL can result in important roles in reducing the active oxygen species by enhancing the fruit antioxidant system in this study. Similar results have also been observed in other studies. Treatment with BRs significantly induced the Vc content and activities of CAT, PAL, PPO and SOD in jujube (Zhu et al., 2010), which is consistent with our results. The exogenous application of BRs enhanced the activity of antioxidant enzymes, including POD, CAT, and glutathione reductase ascorbate and alleviated chilling injury in pepper during storage (Wang et al., 2012). Brassinolide treatment significantly induced the enzymatic activities of PPO, POD, and SOD in cowpea leaves under salt stress, conferring tolerance to salt stress by increasing the activities of those antioxidative enzymes (Elmashad and Mohamed, 2012). Pretreatment of eBL enhanced the antioxidant capacity of wucai *via* inducing the activities of key enzymes of the AsA-GSH including APX, glutathione reductase (GR), dehydroascorbate reductase (DHAR), and monodehydroascorbate reductase (MDHAR), resulting in delayed senescence and quality deterioration (Yuan et al., 2021). All these results are consistent with our results. Other hormones such as salicylic acid and methyl jasmonate also improve fruit physicochemical properties by enhancing the antioxidant activity of mangosteen and dragon fruit during cold storage (Mustafa et al., 2018). The tolerance of plants to stress conditions may be associated with the ability to remove AOS through AOS detoxifying enzymes, such as SOD, GR, POD, CAT, and PPO, indicating that these antioxidant enzymes play an important role in protecting fruit from oxidative damage.

Respiration plays a vital role in high plants, and respiratory metabolism is an important metabolic activity in harvested crops (Lin et al., 2019). Respiratory rate and the respiratory pathways involved are closely related to the ripening and senescence of postharvest horticultural crops (Li et al., 2016). Respiration is one main measurable indicator of the metabolic activity of postharvest crops, with a high respiration rate accelerating ripening or senescence and shortening the storability of fruits and vegetables (Li et al., 2016; Lin et al., 2019). Respiratory metabolic pathways include the EMP, phospho pentose pathway (HMP or PPP), TCA, and CCP (Fernie et al., 2004). The respiratory pathway and respiratory intensity depend on crop varieties, the external environment or different treatment, which are closely related to the changes in activities of respiratory metabolic enzymes (Lin et al., 2018). SDH is the crucial enzyme of TCA pathway, which participates in the reversible oxidation of succinic acid into fumaric acid (Lin et al., 2018). CCO is the key enzyme of CCP, which catalyzes the transfer of electrons from ferrocytochrome *c* to molecular oxygen and plays a key role in aerobic metabolism and energy production during oxidative phosphorylation (Lin et al., 2018). PGI is thought to be the crucial enzyme of EMP, while G-6-PDH and 6-PGDH are the key enzymes involved in PPP (Li et al., 2016). Glucose-6-phosphate (G-6-P) can be transformed to fructose-6-phosphate (F-6-P) through catalysis by PGI in the EMP respiratory pathway. In this study, the four key factors involved in four respiratory metabolic pathways were determined. The content of F-6-P, product of PGI, increased with fruit senescence. Treatment with eBL significantly reduced the production of F-6-P, indicating an inhibition of the EMP pathway. However, treatment with eBL induced the activities of CCO, G-6-PDH, and 6-PGDH compared with the control, indicating that the eBL treatment enhanced PPP and CCP pathways (Figure 5). These results indicated that the decreased activity of EMP respiratory pathway, but the increased ratio of PPP and CCP respiratory pathways, played an important role in delaying carambola fruit senescence following treatment with eBL. It is hypothesized that the treatment with eBL delayed fruit senescence and respiration, which was linked to the inhibition of activities of crucial enzymes in EMP-TCA cycle and the enhancement pf activity of key enzymes in respiratory pathway of PPP and CCP, such as G-6-PDH and 6-PGDH.

These findings were consistent with a previous study that the controlled atmosphere (CA) treatment of 30% O_2_ + 70% CO_2_ accelerated the senescence of broccoli, which was primarily owing to the inhibition in total activities of G-6-PDH, 6-PGDH, CCO, and SDH. However, CA treatment of 50% O_2_ + 50% CO_2_ delayed the senescence of broccoli, which was primarily related to the increased levels of G-6-PDH and 6-PGDH activities and reduced activities of SDH and CCO (Li et al., 2017). In longan fruit, H_2_O_2_ stimulated pulp breakdown owing to a decreased proportion of PPP pathway, the increased proportions of EMP pathway, TCA cycle, and CCP in total respiratory pathways, which primarily regulate the activities of key enzymes, including PGI, SDH, CCO, G-6-PDH, and 6-PGDH (Lin et al., 2020). Salicylic acid (SA) treatment effectively reduced the fruit disease index and respiration rate by decreasing the activities of PGI, SDH, and CCO but boosting the activities of G-6-PDH + 6-PGDH (Chen et al., 2020). This indicated that treatment with SA retards the development of disease *via* decreasing the respiratory pathways of EMP-TCA cycle and CCP but increasing PPP respiratory pathway (Chen et al., 2020). In nectarine fruit, treatment with chitosan delayed fruit senescence, which was primarily owing to the inhibition of the respiration rate and an enhancement of the antioxidant system (Zhang et al., 2019), which is consistent with our results. Additionally, treatment with chitosan significantly suppressed the activity of SDH enzyme and increased total activity of G-6-PDH and 6-PGDH. All of these results showed that the enhancement of PPP and reduction of EMP respiratory pathway aid in the preservation and quality of harvested horticultural crops.

Fruit ripening is a complex process regulated by various factors and coordinated by plant hormones. Hormone cross-talk is critical for the fruit ripening process. There is a close relationship between BR and GA and IAA signaling during many developmental processes in plants (Wang and Yang, 2008). Auxin is a critical hormone in fruit development and IAA is the key regulator of fruit development in non-climacteric and climacteric fruit (Sam et al., 2014). In grape, the highest content of IAA has been detected in flowers and young berries, and it gradually decreases to low levels throughout the ripening period. IAA play a negative role in anthocyanin and sugar accumulation, which associated with delayed ripening of grapeberry (Böttcher et al., 2011; Ziliotto et al., 2012). The auxin contents are usually kept in variant or tend to decrease during the fruit postharvest period. Postharvest exogenous IAA treatments could delay ripening in some fruits (Chen et al., 2016; Moro et al., 2017), thus suggesting auxin also plays an important role in the control of postharvest fruit ripening.

It was reported that exogenous brassinosteroid treatments increased growth and endogenous brassinosteroid, auxin, as well as gibberellin levels of apple nursery trees (Zheng et al., 2019). However, brassinosteroids and gibberellic acid (GA3) play different roles in cherry fruit coloring process, and brassinosteroids promoted fruit coloring but GA3 sprays significantly delayed maturation time and the development of skin color (Li et al., 2020). ABA play an important role in fruit ripening, especially for non-climacteric fruit. Studies have found that BRs and ABA interact to regulate many biological processes in plants (Zhang et al., 2009). ABA is important for seed dormancy during embryo maturation and inhibits seed germination, but BRs promote seed germination, which tend to antagonize the effect of ABA (Finkelstein et al., 2008). A study also found that BR treatment promoted the production of ethylene and IAA in squash hypocotyls but reduced the ABA content (Jong-Seon et al., 1989).

For the non-climacteric fruit such as grape and strawberry, the content of IAA and GA decreased with fruit ripening. In addition, the content of ABA and ethylene increased with fruit ripening (Fuentes et al., 2019). This results also observed in carambola fruit in present work. Our results showed that the contents of IAA and GA3 decreased with fruit ripening in carambola fruit, but eBL treatment delayed fruit ripening and increased the content of IAA and GA3. ABA content increased with fruit ripening but reduced by eBL treatment. All these results seem that IAA and GA are negatively related to fruit ripening of carambola fruit and ABA is positively related to carambola fruit ripening. BRs interact with these hormones and affects their synthesis and coregulates carambola fruit ripening process. However, the underline cross-talk regulation needs further investigation.

## Conclusion

This study showed that treatment with eBL delayed the ripening of carambola fruit and improved its taste by reducing content of organic acids. Treatment with eBL inhibited the respiration rate of fruit and enhanced antioxidant system by increasing the activities of POD, SOD, CAT, and AAO and decreasing the accumulation of H_2_O_2_ and MDA. eBL alters the respiratory metabolic pathway by inhibiting activity of PGI but increasing activity of G-6-PDH and 6-PGDH and CCO. The endogenous hormone contents of fruit were altered by application of eBL. The contents of IAA and GA were induced, but that of ABA was reduced. In general, treatment with eBL primarily maintains the quality of carambola fruit by enhancing the antioxidant capacity of fruit and altering its respiratory metabolism. The increased PPP and CCP respiratory pathway and decreased EMP pathway may aid in the preservation of carambola fruit.

## Data Availability Statement

The original contributions presented in the study are included in the article/supplementary material, further inquiries can be directed to the corresponding author/s.

## Author Contributions

XZ: conceptualization, funding acquisition, resources, and writing—original draft. YC: investigation, data curation, formal analysis, and methodology. JL: software, investigation, and methodology. XD: resources and validation. SX: software and methodology. SF: software, methodology, and validation. ZS: methodology and validation. WC: supervision, resources, and writing—review and editing. XL: conceptualization, funding acquisition, project administration, resources, supervision, and writing—review and editing. All the authors read and approved the final manuscript.

## Conflict of Interest

The authors declare that the research was conducted in the absence of any commercial or financial relationships that could be construed as a potential conflict of interest.

## Abbreviations

eBL: 2,4-epibrassinolide
BRs: brassinosteroids
6-P-F: fructose-6-phosphate
CCO: cytochrome oxidase
G-6-PDH: glucose-6-phosphate dehydrogenase
6-PGDH: 6-phosphate gluconate dehydrogenase
AsA: ascorbic acid
PVP: polyvinylpyrrolidone
MDA: malondialdehyde
PPO: polyphenol oxidase
POD: peroxidase
CAT: catalase
SOD: superoxide dismutase
AAO: ascorbic acid oxidase
PGI: hexose isomerase
SDH: succinate dehydrogenase
ABA: abscisic acid
GA: gibberellin acid
IAA: indole-3-acetic acid
AOS: active oxygen species.
